# Laparoscopic Radical Cystectomy in the Elderly – Results of a Single Center LRC only Series

**DOI:** 10.1590/S1677-5538.IBJU.2015.0419

**Published:** 2016

**Authors:** Tom J. N. Hermans, Laurent M. C. L. Fossion, Rob Verhoeven, Simon Horenblas

**Affiliations:** 1 Department of Urology, Maxima Medical Center Veldhoven, Veldhoven, Netherlands; 2 Eindhoven Cancer Registry/Comprehensive Cancer Centre South, Eindhoven, Netherlands; 3 Department of Urology, The Netherlands Cancer Institute, Antoni van Leeuwenhoek Hospital, Amsterdam, Netherlands

**Keywords:** Cystectomy, Geriatrics, Minimally Invasive Surgical Procedures, Survival, Urinary Bladder Neoplasms

## Abstract

**Objective:**

To compare outcome of laparoscopic radical cystectomy (LRC) with ileal conduit in 22 elderly (≥75 years) versus 51 younger (<75 years) patients.

**Materials and Methods:**

Analysis of prospectively gathered data of a single institution LRC only series was performed. Selection bias for LRC versus non-surgical treatments was assessed with data retrieved from the Netherlands Cancer Registry.

**Results:**

Median age difference between LRC groups was 9.0 years. (77.0 versus 68.0 years). Both groups had similar surgical indications, body mass index and gender distribution. Charlson Comorbidity Index score was 3 versus 4 in ≥50% of younger and elderly patients. Median operative time (340 versus 341 min) and estimated blood loss (<500 versus >500mL) did not differ between groups. Median total hospital stay was 12.0 versus 14.0 days for younger and elderly patients. Grade I-II 90-d complication rate was higher for elderly patients (68 versus 43%, p=0.05). Grade III-V 90-d complication rate was equal for both groups (23 versus 29%, p=0.557). 90-d mortality rate was higher for elderly patients (14 versus 4%, p=0.157). Median follow-up was 40.0 months for younger and 57.0 months for elderly patients. Estimated overall and cancer-specific survival at 5years. was 46% versus 35% and 64% versus 64% for younger and elderly patients respectively.

**Conclusions:**

Our results suggest that LRC is feasible in elderly patients, where a non-surgical treatment is usually favoured.

## INTRODUCTION

Bladder cancer (BC) rates are highest in people aged 75-84 years old (years), with a median age of 73 at diagnosis (1). Since life expectancy is still rising in Europe and the United States a decline in the incidence of BC in the elderly is not to be expected (2, 3). Open radical cystectomy (ORC) is the reference standard in the treatment of muscle invasive bladder cancer (MIBC) (4). Whether this surgical approach should also be used in the elderly is an ongoing debate (5–7). Non-surgical approaches, such as (chemo) radiation, are considered an alternative in frail or elderly patients (6). Minimal invasive procedures such as laparoscopic radical cystectomy (LRC) might, also in elderly patients, tilt the balance to surgery (8–12). We assessed morbidity and mortality in elderly patients undergoing LRC with ileal conduit and compared outcomes for younger (<75years) and elderly (≥75years) patients.

## PATIENTS AND METHODS

From September 2006 until February 2013, 80 consecutive patients underwent LRC with a standard pelvic lymph node dissection (PLND) at Maxima Medical Center, Veldhoven, the Netherlands. Indications included: (1) MIBC cT2–4a, cN0-1, Nx, cM0; (2) BCG-resistant high-risk and recurrent non-MIBC (Tis, T1G3), and (3) extensive papillary disease that could not be controlled with transurethral resection and intravesical therapy alone. Patients who underwent salvage cystectomy (n=1) or received urinary diversions other than ileal conduit (n=6) were excluded. Seventy-three patients were included. To evaluate possible selection bias for surgery vs. other treatment modalities, data concerning primary treatment modalities applied, age and comorbidity for all patients diagnosed with ≥cT1-4 N0-3, x M0-1, x BC at our hospital within the study period were retrieved from the Netherlands Cancer Registry. This nationwide registry obtains notification of cancer diagnoses via PLAGA (the national network and registry of histopathology and cytopathology in the Netherlands) and independent clerks collect data from patient files.

This registration does not apply the Charlson Comorbidity Index (CCI), but scores one point per category if cardiovascular disease, hypertension, pulmonary disease, diabetes or a previous malignancy is present. There was no selection between ORC and LRC, since only LRC was performed at our hospital. The surgical technique used at our hospital has been described previously (13). A single surgeon performed the first 53 procedures and during the remainder of the procedures another surgeon was trained by the principle surgeon (only small steps, e.g. lymph node dissection, ileal conduit). During the study period the principle surgeon performed a total of 630 laparoscopic oncological procedures. First follow-up visit was 6 weeks after the procedure and at 6-month intervals including medical history, physical examination and routine hematological and biochemical laboratory examination. Abdominal ultrasonography and chest X-ray were performed every 6 months and a chest and abdomen CT-scan annually, unless otherwise clinically indicated. Follow-up was conducted at least for 90 postoperative days in our own center.

An arbitrary cut-off age of ≥75years. was used to define elderly patients (n=22) (5, 14). Baseline characteristics, perioperative and follow-up data were gathered prospectively and analyzed retrospectively. The following perioperative parameters were assessed: operative time, estimated blood loss (EBL), the number of blood transfusions, conversions, intensive care unit admissions, total length of hospital stay (LOS) and 30- and 90-d mortality. Operative time was defined as the time from incision to final closure, blood loss was estimated via collection of the suction device and LOS was calculated as the time from surgery until clinical discharge in days. Postoperative complications within 90 days after surgery were classified according to the modified Clavien system and were retrospectively registered from patient files and prospectively at follow-up visits by the second author (surgeon) of this manuscript (15). Overall survival (OS) and Cancer specific survival (CSS) were assessed for all patients as to July 2015. Survival times were defined as the time elapsed from LRC to the date of death (OS) or death of bladder cancer (CSS). Statistical analyses were performed by using SPSS statistical software (version 19.0; SPSS Inc., Chicago, Ill., USA). Survival was estimated with the Kaplan-Meier method and the Log-rank test to compare survival between groups. Values were considered statistically significant at p<0.05.

## RESULTS

Patient characteristics are presented in Table-1. Twenty-two (30%) out of 73 patients were ≥75years. The median age difference compared to patients <75years. was 9.0 years. (77.0 vs. 68.0, p<0.001). There were no significant differences regarding gender, Body Mass Index (BMI), surgical indications and neoadjuvant treatments between groups. The CCI score in ≥50% of patients was one point higher in the elderly (3 vs. 4, p<0.001), even when points attributed for age were distracted (0 vs. 1, p=0.028). Regarding total BC population, patient's aged ≥75years. who underwent cystectomy were significantly younger than patients who underwent other treatment modalities (p<0.001), but had similar comorbidity scores (p=0.334) as scored by the Nederlands Cancer Registry, Table-2. In total, 50% of patient's ≥75years. with cT2-4N0M0 disease and a comorbidity score of 2.0 in ≥50% underwent cystectomy. This comorbidity score was equal for patients who underwent other treatment modalities or no treatment (p=0.299).

**Table 1:** Pre-, per-, and postoperative patient characteristics.

		Total N = 73	< 75 years N = 51	≥ 75 years N = 22	P-value
**Gender, n (%)**					
	Male	59 (80)	42 (82)	17 (77)	0.613^a^
	Female	14 (19)	9 (18)	5 (23)	
**Age** (year), median (range)		70.1 (42–86)	68.0 (42–74)	77.0 (75-86)	<0.001^b^
BMI^e^ (kg/m^2^), median (range)		25.7 (16.0-37.6)	25.4 (16.0-37.6)	26.4 (17.3-36.0)	0.799^c^
CCI^f^ (age included), (range)		≥3.0 in 50% of	≥3.0 in 50% of	≥4.0 in 50% of	<0.001^b^
		patients (0-8)	patients (0-5)	patients (3-8)	
CCI^f^ (age score excluded), (range)		≥0.0 in 50% of	≥0.0 in 50% of	≥1.0 in 50% of	0.028^b^
		patients (0-5)	patients (0-3)	patients (0-5)	
**Indication, n (%)**					
	NMIBC^g^	16 (22)	9 (18)	7 (32)	0.179^a^
	MIBC^g^	57 (78)	42 (82)	15 (68)	
Neoadjuvant chemotherapy, n (%)		2 (3)	2 (4)	0 (0)	1.0^d^
Operative time (min), median (range)		340 (260-780)	340 (260-510)	341.0 (271-780)	0.852^b^
Estimated blood loss (mL), median (range)		500 (100-3300)	500 (100-3300)	500 (100-2000)	0.346^b^
Pre-operative Hemoglobin (mmoL/L) median (range)		8.4 (5.1-10.3)	8.8 (5.10-10.3)	7.6 (6.0-7.6)	0.004^c^
Transfusions during hospital stay, n (%)		21 (29)	10 (20)	11 (50)	0.01^a^
Conversions, n (%)		0 (0)	0 (0)	0 (0)	>0.05^d^
Intensive care unit admissions, n (%)		16 (22)	10 (20)	6 (27)	0.468^a^
Total hospital stay (days), median (range)		13.0 (6-147)	12.0 (6-147)	14.0 (8-28)	0.133^b^
**Mortality, n (%)**					
	30 days	1 (1)	1 (2)	0 (0)	1.0^d^
	90 days	5 (7)	2 (4)	3 (14)	0.157^d^
**Complications 90-days n (%)**					
	Clavien I/ II (incl. transfusions)	37 (51)	22 (43)	15 (68)	0.05^a^
	Clavien I/ II (excl. transfusions)	26 (36)	15 (29)	11 (50)	0.092^a^
	Clavien III/ IV/ V	20 (27)	15 (29)	5 (23)	0.557^a^

a Chi-square test;

b Mann-Whitney U test;

c Independent sample T test;

d Fischer's exact test;

e Body Mass Index;

f Charlson Comorbidity Index;

g (N) MIBC = (Non) Muscle Invasive Bladder Cancer;

h One or more complications per patient per category (Clavien grade I-II or III-V)

**Table 2:** Primary treatment for cT1-4 N0-3,x M0-1,x bladder cancer patients at our hospital from September 2006 until February 2013 in relation to age and comorbidity. Data derived from the Netherlands Cancer Registry.

Overall (n=208)					
	Cystectomy	Radiotherapy and/ or chemotherapy	Transurethral resection and/ or intravesical therapy	No treatment	Unknown
**N (%)**	77 (37)	30 (14)	90 (43)	9 (4)	2 (1)
**Age year** a **(median)**	69.0	74.0	74.0	84.0	70.0
**Comorbidity score** b	≥1.0 in 50% of patients	≥2.0 in 50% of patients	≥1.0 in 50% of patients	≥3.0 in 50% of patients	-
Patients aged ≥ 75 years (n=85)					
	Cystectomy	Radiotherapy and/ or chemotherapy	Transurethral resection and/ or intravesical therapy	No treatment	Unknown
**N (%)**	22 (26)	13 (15)	44 (52)	6 (7)	-
**Age year** c **(median)**	77.5	85.0	82.0	87.5	
**Comorbidity score** d	≥2.0 in 50% of patients	≥2.0 in 50% of patients	≥2.0 in 50% of patients	≥2.5 in 50% of patients	-

a Independent sample T-test for age at cystectomy vs. radiotherapy and/ or chemotherapy p=0.016;

b Independent sample T-test for comorbidity at cystectomy vs. radiotherapy and/ or chemotherapy p=0.008;

c Mann-Whitney U test for age at cystectomy vs. radiotherapy and/ or chemotherapy p<0.001;

d Mann-Whitney U test for comorbidity at cystectomy vs. radiotherapy and/ or chemotherapy p=0.334

**Note:** Comorbidity was scored for: cardiovascular disease, hypertension, pulmonary disease, diabetes and previous malignancy. If present, 1 point per category was scored. Please note, this is not the Charlson Comorbidity Index Score.

Perioperative outcome parameters are presented in Table-1. While median operative time and EBL did not differ significantly between groups, the percentage of patients that received a blood transfusion differed (20 vs. 50%, p=0.006). The median pre-operative hemoglobin level was however significantly lower in the elderly (8.8 vs. 7.6mmoL/L, p=0.004). In both groups no conversions were needed. The percentage of intensive care unit admissions and median LOS were not significantly different between groups. One patient <75years. died within 30 days after surgery vs. none of the elderly patients (p=1.0). Within 90 days in total 2 and 3 patients died in the younger and elderly group, respectively (p=0.157). The number of patients who experienced ≥1 treatment related grade I-II complication within 90 days after surgery was higher in the group of elderly patients (68 vs. 43%, p=0.05). This in contrast to the grade III-V complication rate, which was equal in both groups (29 vs. 23%, p=0.557). Complications per patient group are shown in Table-3.

**Table 3:** complications within 90 days after laparoscopic radical cystectomy for younger (<75 years) and elderly (≥75 years) patients according to the modified clavien classification system.

<75 years (N=51)			
Clavien grade	Complication	Cases (N)	Management
Peroperative			
I	Damage to iliac vein	1	Laparoscopic suturing
I	Rectal lesion	3	Laparoscopic suturing
Iva	Rupture of lumbar vein	1	Laparoscopic suturing
≤ 90 days			
I	Anemia	10	Blood transfusion
I	Delirium	1	Antipsychotics
I	Wound infection	2	Antibiotics and bedside management
II	Acute myocardial infarction	1	Pharmacological treatment
II	Deep venous thrombosis	1	Pharmacological treatment
II	Exacerbation COPD	1	Pharmacological treatment
II	Pneumonia	4	Antibiotics
II	Prolonged bowel ileus (no acceptance of oral intake 5 days after surgery)	6	Conservative
II	Urinary tract infection/ sepsis	4	Antibiotics
IIIa	Ureteroileal obstruction with or without infection	2	Nephrostomy (and antibiotics)
IIIa	Lymphatic leakage with or without infection	2	Percutaneous drainage
IIIa	Necrosis of praeputium penis	1	Surgery
IIIb	Compartment syndrome lower leg	2	Surgery
IIIb	Iatrogenic corpus alienum (drain) in the abdomen	1	Surgery
IIIb	Iatrogenic defect ileal conduit after correction incisional hernia	1	Surgery
IIIb	Iatrogenic pneumothorax	1	Surgery
IIIb	Incisional hernia (+ after relaparotomy)	3	Surgery
IIIb	Rectourethral fistula	2	Surgery
Iva	Acute tubular necrosis	1	Haemodialysis
Iva	Ileal anastomotic leakage	2	Surgery
IVb > V	Pneumosepsis	1	-
IVb > V	Candida sepsis	1	-
≥75 years (N=22)			
Peroperative			
I	Rectal lesion	1	Laparoscopic suturing
I	Damage to iliac vein	1	Laparoscopic suturing
≤ 90 days			
I	Anemia	11	Blood transfusion
I	Delirium	1	Antipsychotics
I	Temporary paresis of right upper arm	1	Conservative
I	Wound infection	1	Antibiotics and bedside management
II	Atrial flutter	1	Pharmacological treatment
II	Pneumonia	3	Antibiotics
II	Prolonged bowel ileus (no acceptance of oral intake 5 days after surgery)	2	Conservative
II	Urinary tract infection/ sepsis	2	Antibiotics
IIIa	Uretero-ileal obstruction with or without infection	4	Nephrostomy (and antibiotics)
IIIa	Lymphatic leakage with or without infection	1	Percutaneous drainage
IIIb	Rectourethral fstula	1	Surgery
Iva	Rabdomyolysis	1	Conservative

Please note that some patients had more than one complication, which were all registered separately. E.g. pneumonia, delirium and prolonged bowel ileus in one patient.

Pathology results are presented in Table-4. In both groups main histologic tumor type was urothelial carcinoma. Non-organ confined disease (≥pT3) was slightly more prevalent in the elderly (45 vs. 55%, p=0.458), as was the percentage of patients with lymph node involvement (29 vs. 32%, p=0.558) and the percentage of positive surgical margins (PSM) (8 vs. 23%, p=0.118). The mean numbers of lymph nodes removed were 15.7 and 13.3, respectively. Median overall follow-up was 40.0 months for younger and 57.0 months for elderly patients. OS and CSS were equal for both groups (Log rank p-value of 0.668 and 0.868, respectively). Estimated OS and CSS at 5years. was 46% vs. 35% and 64% vs. 64% for younger and elderly patients, respectively. Kaplan-Meier curves are presented in Figure-1.

**Table 4:** Pathology results.

		Total N = 73	< 75 years N = 51	≥ 75 years N = 22	P-value
**Histology, n (%)**					
	Urothelial carcinoma	71 (98)	50 (98)	21 (96)	0.515^a^
	Squamous cell carcinoma	1 (1)	0 (0)	1 (4)	
	Bladder sarcoma	1 (1)	1 (2)	0 (0)	
**Organ confining, n (%)**					
	Organ-confined disease (≤ pT2)	38 (52)	28 (55)	10 (45)	0.458^b^
	Non organ confned disease (>pT3)	35 (48)	23 (45)	12 (55)	
Lymph nodes removed, mean		14.9 (1-36)	15.7 (1-32)	13.3 (3-36)	0.063^c^
**Lymph node involvement, n (%)**					
	pN0	50 (69)	35 (68)	15 (68)	
	pN1	9 (12)	5 (10)	4 (18)	0.558^b^
	pN2 or N3	13 (18)	10 (20)	3 (14)	
	unknown	1 (1)	1 (2)	0 (0)	
Positive surgical margins, n (%)		9 (12)	4 (8)	5 (23)	0.118^a^
Median follow-up, months (range)		46.0 (0-97)	40.0 (0-97)	57.0 (1-75)	0.445^c^

a Fischer's exact test;

b Chi-square test;

c Mann-Whitney U test

**Figure 1:**
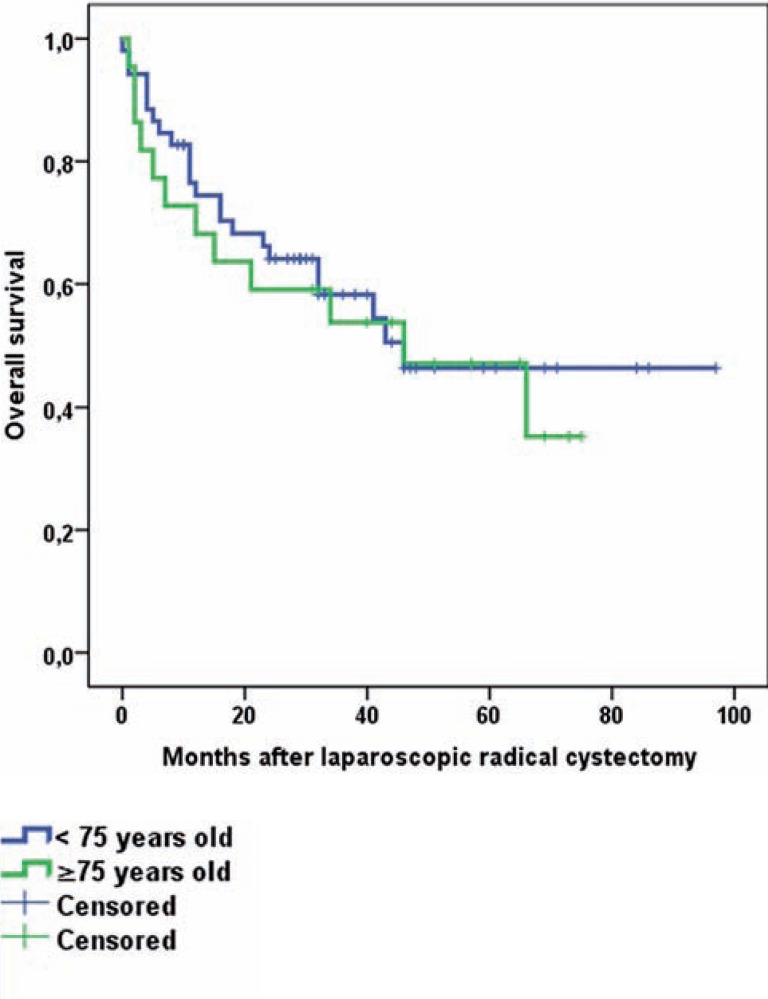
A) Overall survival for laparoscopic radical cystectomy for younger vs. elderly.

**Figure 1:**
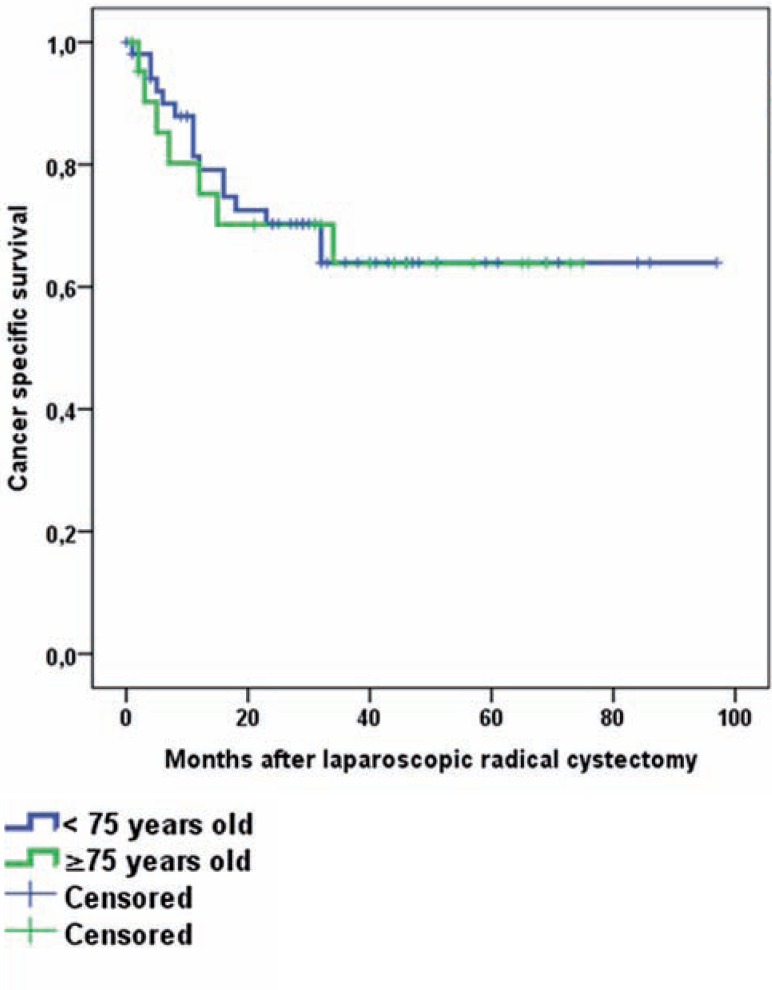
B) cancer specific survival for laparoscopic radical cystectomy for younger vs. elderly.

## DISCUSSION

ORC is considered the gold standard in the treatment of high-risk non-MIBC and MIBC (4). Analysis of currently outdated 1992 Surveillance, Epidemiology, and End Results (SEER) data showed RC was only performed in 14% of patients with MIBC aged >75years. Moreover, patients <75years., classified ASA 1-2 or ASA 3-4 were 3 to 12 times more likely to undergo radical cystectomy (RC) than those ≥75years. (16). More recent linked SEER and Medicare data (1992-2002) showed a slight increase with RC performed in 21% (17). In the Netherlands, RC is performed in less than 30% of patients aged ≥>75years. with clinical stage II-III BC (18%), in our cohort this was 50% (18). In a cohort of 3262 patients with MIBC aged >65years., elder age at diagnosis (>80 vs. 66-69years), a higher CCI score (3 vs. 0-1) and a long travel distance to an available surgeon (>50 vs. 0-4 miles) were significantly associated with decreased chance of undergoing RC (17). The 5-year adjusted OS and CSS rates for cystectomy vs. chemotherapy and/or radiation or surveillance were 42%, 21%, 15% and 67%, 48%, 43%, respectively; however, this was never assessed in a prospective randomized fashion (18). The SEER-database study of Chamie et al. showed a 6 and 20 months OS and CSS benefit in the very elderly (≥80years) undergoing RC with PNLD compared to those receiving bladder sparing treatments with radiotherapy (19). In the group of elderly actually undergoing RC, further important predictors of OS are performance and nutritional status. In a group of 152 patients aged ≥70years., a Karnofsky Performance Status (KPS) of 90-100 was associated with a 9-month OS benefit compared to a KPS <80, irrespective of chronological age (20). Poor nutritional status can increase 90-d mortality even up to 17% (21). The Eindhoven Cancer Registry data (1995-2009) of 2445 patients with MIBC underline before mentioned statistics, since also in our region RC was significantly associated with better OS, independently of age, socioeconomic status and serious comorbidity (22). The above-mentioned study results have to be interpreted with great caution since randomized controlled trials between RC and (multimodal) organ-preserving therapies are lacking.

Exact reasons for withholding RC in the elderly are unknown, but a combination of biological and quality of life factors is most likely. A recent multicenter validation study on the prognostic value of patient age in RC with PLND involving 4429 patients (1979-2008) showed that higher age was significantly associated with advanced pathologic tumor stages, higher tumor grades, lymph node invasion and a higher number of PSMs. This trend was also seen in our elderly population (Table-3). In categorical multivariable regression analyses adjusted for tumor grade, tumor stage, margin status, lymph node status, lymphovascular invasion and adjuvant chemotherapy, only age >80years. was significantly associated with worse CSS and OS rates compared to the younger. OS was also significantly worse for those aged >60years., remarkably CSS was not, which is in line with our oncological outcome results (23).

Another possible barrier is the urologist, who is reluctant to perform the operation and is unwilling to refer patients to someone who is (24). It is the high complication and mortality rates which may discourage surgeons from performing RC in the elderly (5, 7, 16, 17, 19). Not unreasonable considering complication rates of 30-60% (25, 26). In a center of excellence even 64% of patients, regardless of age, developed at least one complication within 90 days after surgery (27). Sound comparison between centers is however hardly possible due to heterogeneous definitions of complications (5). A recently published randomized controlled trial of open, laparoscopic and robotic radical cystectomy reported a significantly lower 30-d complication rate in the LRC group than in the ORC group (12). The 90-d complication rate showed a trend towards fewer complications in the LRC group (12). These findings may be of special importance in frail elderly patients. In our series elderly were more prone to develop grade I-II (68 vs. 43%), but not grade III-IV complications (23 vs. 29%). This is in line with the study results of Guillotreau et al. (28). They studied 146 patients who underwent minimal invasive RC (131 LRC's) and showed grade I-II and grade III-V 90-d complication rates of 71 vs. 89% and 29 vs. 10% for younger (<70years) vs. elderly (≥70years) patients, respectively. Zeng et al. reported grade I-II and grade III-V 90-d complication rates in 38 vs. 92% and 5 vs. 16% for 21 LRC vs. 25 ORC patients aged >75years., respectively (29). In our study no remarkable differences in type of complications were seen between groups, except for the higher percentage of uretero-ileal obstructions in the elderly (6 vs. 18%), which warrants further investigation.

Perioperative mortality is an important indicator of surgical quality and might indicate the feasibility of novel techniques. The 14% 90-d mortality rate in our elderly population can be considered high, but patients did not decease due to surgery itself and resection margins were clean (Table-5). Moreover, population-based studies report 90-d mortality rates after RC in the elderly of 9 up to 15% (5, 7, 30). Recently, the European Association of Urology (EAU)-section of Uro-technology (ESUT) reported comparable long-term oncological outcome for LRC (N=503) and contemporary ORC series (8). Selection bias for LRC vs. ORC and LRC vs. other treatment modalities in elderly could, in contrast to our series, not be excluded. The ESUT study included, among others, all patients presented in the current manuscript. Sub analysis for elderly patients was however not conducted (8). Extending the 90-d follow-up period in our series did not significantly change OS and CSS rates for elderly vs. younger patients (Figure-1), independent of the median age difference of 9.0 years, worse CCI-scores adjusted for age and worse pathological outcome results, Table-4. Since PSM-status is one of the most important predictors of survival and in general is present in 6-7% of patients treated with RC, our PSM-status in 11% of the total study population is a point of concern (22, 31). However, 78% of patients with a PSM in our population had non-organ confined disease. Altogether the study results of our LRC series are not completely in line with the systematic review results of Froehner et al. who concluded that next to mortality, morbidity is also increased in elderly patients undergoing ORC (5).

**Table 5:** characteristics of patients who died within 90 days after surgery.

	Age	CCI^a^	Pathology	Period to death	Cause of death
1	77	6	pT3N1 R0	< 90-d	Metastatic disease
2	79	4	pT3N1 R0	< 90-d	Gastro-enteritis with dehydration
3	80	5	pT3N2 R0	< 60-d	Metastatic disease
4	67	3	pT2N0 R0	< 30-d	Postoperative pneumosepsis
5	70	3	pT4N1 R1	< 60-d	Metastatic disease

a Charlson Comorbidity Index

Morbidity is especially related to the urinary diversion, but no randomized controlled studies comparing ileal conduits with neobladder diversions have been performed (4). However, since it is estimated that complication rates for neobladder diversions are higher than for ileal conduits we excluded patients with a neobladder diversion from analyses. Being all younger, results would be biased in favor of elderly patients. Due to limited experience with neobladder diversions, only 11% of the younger patients in our series received a neobladder diversion. Currently, guidelines state it is not possible to recommend a particular type of diversion (4).

In contrast to ORC, outcome data for LRC in the elderly is still sparse (28, 29). From aforementioned preliminary retrospective studies, it seems that in experienced hands minimal invasive surgery for MIBC in the elderly is feasible (28, 29). However, all studies so far lack insight in total bladder cancer population and the distribution of the given treatment modalities in relation to age, comorbidity scores, nutritional and performance status. Altogether selection bias for the application of minimal invasive techniques cannot be excluded. For the near future improvements in the above mentioned factors, results of controlled trials and confirmation of long-term oncological outcome results have to be waited before more definitive conclusions can be drawn and to accept a more widespread application of minimal invasive surgery for MIBC in younger and elderly patients.

Our study results are subjected to the limitations and biases of retrospective analyses of a prospective database and the small number of elderly patients. Selection bias for surgery in the elderly cannot be excluded, but seems to be relatively small regarding the distribution of applied treatment modalities in total bladder cancer population in relation to age and comorbidity (Table-2). Moreover, 50% of patients in our cohort aged ≥75years. with clinical cT2-4N0M0 BC underwent radical cystectomy. An important limitation is the absence of an ORC control group. This study, in contrast to many others, however excludes selection bias between LRC and ORC and shows comparable outcome result for elderly vs. younger patients undergoing LRC. Based on the aforementioned RCT results of Khan et al. (12) and our data we assume that minimal invasive procedures may tilt the balance to surgery in elderly patients.

## CONCLUSIONS

Our results suggest that LRC is feasible in elderly patients, where a non-surgical treatment is usually favoured.
